# Supplementary material for: Fabrication and Characterization of Co-Sensitized Dye Solar Cells Using Energy Transfer from Spiropyran Derivatives to SQ2 Dye
Source: Molecules. 2024 Oct 16;29(20):4896. doi: 10.3390/molecules29204896 (PMC11510412; doi:10.3390/molecules29204896)
Supplement: Supplementary file 1 [file molecules-29-04896-s001.zip › molecules-3226825-supplementary.pdf]

molecules

MDPI

Supplementary data for

Fabrication and characterization of co-sensitized dye solar cells using energy transfer from spiropyran derivatives to SQ2 dye

Michihiro Hara [1*] and Ryuhei Ejima

Department of Applied Science and Engineering, Fukui University of Technology, Fukui, Japan

* Correspondence: hara@fukui-ut.ac.jp; Tel: +81-776-29-2446 (M. Hara)

1. Molar absorption coefficient spectrum and Emission spectrum of **SQ2** Solution in ethanol

In the UV-visible molar extinction coefficient spectrum of **SQ2** (3.0×10^{-6} M) in ethanol, maximum absorption wavelengths and shoulders were observed at 654 nm (ε :3.1 × 105 M) and 610 nm (ε:7.1×10^{4} M) (black line in Fig. S1). Fluorescence spectrum was observed at 674 nm under 654 nm irradiation (red line in Fig. S1)

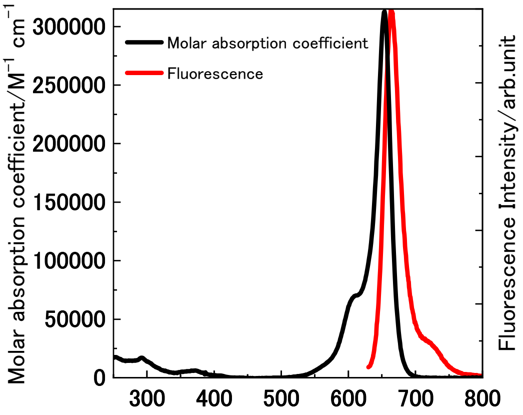

Fig. S1 the UV-visible molar extinction coefficient spectrum (black line) and fluorescence (red line) spectrum of **SQ2**(3.0×10-6 M) dye in ethanol under excitation at 654 nm.

2. Absorbance spectra and Emission spectrum of **SPNO₂** Solution in ethanol

$SPNO_2$ in ethanol showed a absorption in the UV region (black line in Figure S2), and color-less.

Therefore, absorption peak at visible region was assigned to the formed **PMC** by spontaneous isomerization of **SPNO2** solution.Absorption peak of **PMC** at ~540 nm had increasing after UV light irradiation (red line in Fig. S2), and the solution color changed to the red. Fluorescence spectrum peak was observed at 633 nm un-der 540-nm irradiation (orange line in Fig. S2). On the hand, the absorption spectrum of the **PMC** solution after visible light irradiation for 2 minutes was observed in the range of 250 nm-400 nm(green line in Fig. S2), similar to the **$SPNO_2$** solution(black line) .

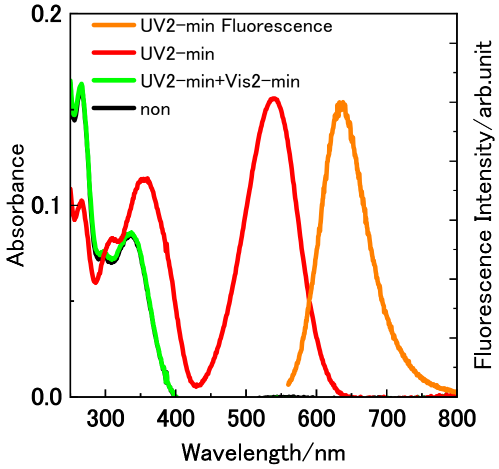

Fig. S2 Absorbance spectra of **$SPNO_2$**(9.1×10^{-6} M) in ethanol(black line) using UV(red line) and visible(green line) light irradiations. Emission spectrum of **$SPNO_2$**(9.1×10^{-6} M) in ethanol under excitation at 540 nm(orange).

3. Time-resolved fluorescence decay curves for **PMC** electrode and **SQ2/PMC** electrode.

Figure S3 shows the time-resolved fluorescence decay curves for the **PMC** electrode (black line) and the **SQ2/PMC** electrode (red line). Overlaying the two graphs shows that the **SQ2/PMC** electrode decays faster than the **PMC** electrode. This suggests that the fluorescence lifetime of **PMC** is shorter at the **SQ2/PMC** electrode than at the **PMC** electrode. The mechanism for the short fluorescence lifetime of the **SQ2/PMC** electrode was thought to be due to the energy transfer from **PMC** to **SQ2**.

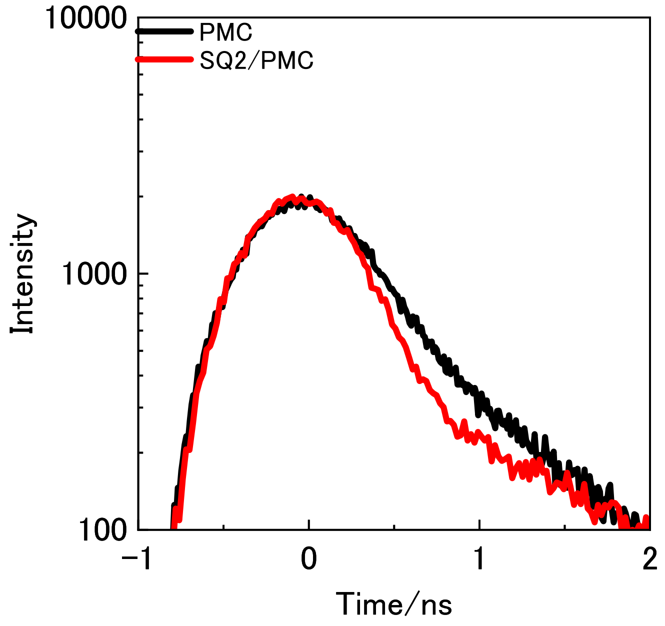

Fig. S3 Time-resolved fluorescence decay curves for the **PMC** electrode (black line) and the **SQ2/PMC** electrode (red line).
